# Therapeutic singing and expiratory muscle strength training in Parkinson's disease: a mixed methods comparison

**DOI:** 10.3389/fresc.2024.1478490

**Published:** 2024-11-21

**Authors:** Jessy Brown, Elizabeth L. Stegemöller

**Affiliations:** Neuroscience and Gerontology Program, Department of Kinesiology, Iowa State University, Ames, IA, United States

**Keywords:** Parkinson's disease, singing, respiratory, mixed methods, expiratory muscle strength training

## Abstract

**Introduction:**

The purpose of this study was to understand how two respiratory strengthening protocols, therapeutic singing (TS) and expiratory muscle strength training (EMST), compare on measures of quality of life (QOL), depression and anxiety for persons with Parkinson's disease. An equally important aim was to understand participants' perceptions of both treatments.

**Methods:**

Quantitative and qualitative datasets were integrated in a convergent mixed methods design within a randomized crossover intervention trial. Thirteen persons with mild-moderate PD (Hoehn and Yahr stage 1–3) completed both interventions, in random order, for 4 weeks, 5 days per week, for approximately 20 min per day. Participants completed self-report questionnaires (Geriatric Depression Scale, Parkinson's Anxiety Scale, Parkinson's Disease Questionnaire-39, and a Survey after Treatment) after each intervention, and twelve participants' qualitative data were analyzed.

**Results:**

Quantitative data did not reveal significant differences between the interventions in depression on the Geriatric Depression Scale or anxiety on the Parkinson's Anxiety Scale and the qualitative data support those findings. There were no significant differences between interventions in QOL as measured by the Parkinson's Disease Questionnaire-39, but there was a main effect of time, with a significant decline (*p* = 0.01) in perceived QOL between baseline and the final visit. The quantitative data diverged from the qualitative data as there were no themes that emerged to corroborate a decrease in QOL. Five qualitative themes were derived from thematic analysis: Benefits, Accessibility, Acceptability, Advice/Feedback, and Preference. Participants' perceptions of the interventions were closely aligned to individual differences and preferences, with an equal split of participants preferring TS and EMST.

**Conclusions:**

Findings from this mixed methods comparison of two respiratory interventions will help to improve the acceptability and accessibility of the interventions to better facilitate adherence to the interventions and promote continued engagement, thereby delaying respiratory decline in those with PD.

## Introduction

Parkinson's disease (PD) is an age-related neurodegenerative disease, and its prevalence is increasing (1). It is estimated that most persons with PD (PwPD) experience respiratory disorders, up to 67% for upper airway obstructions and up to 98% for restrictive changes, and ultimately up to 70% experience aspiration pneumonia associated mortality (2). Respiratory symptoms, which can impair breathing and the ability to cough, can also significantly affect psychosocial well-being, often leading to issues such as depression and anxiety (3). Depression and anxiety are common non-motor symptoms that impact quality of life (QOL) during all stages of the disease (4). Since QOL is influenced greatly by levels of depression and anxiety, an improvement in these outcomes may lead to more adherence to treatment protocols for respiratory dysfunction (5).

Pharmaceutical options to treat respiratory disorders have not shown efficacy, and there are limited non-pharmaceutical treatment options which include respiratory muscle strength training (6), and sensorimotor training for airway protection for cough-related outcomes (7). Expiratory muscle strength training (EMST) is considered the gold-standard for respiratory care for respiratory outcomes in PD. It is a strength-based technique that uses expiratory resistance training to increase the ability of the expiratory muscles to generate more force and contraction during activities such as coughing. Therapeutic singing (TS) is an alternative treatment option shown to have positive outcomes on respiratory measures after singing in PwPD (8–10). Although TS is an emerging and popular evidence-based approach, its effectiveness compared to more established interventions like EMST in terms of respiratory outcomes, patient perceptions, and psychosocial factors such as QOL, depression, and anxiety – key determinants of adherence and long-term engagement – remains unknown.

Little research has been allocated to investigating the acceptability of EMST or its impacts on QOL for those with PD. One study measured QOL before and after EMST and a sham group (11). Results indicated that both groups had increased scores on the swallowing-specific assessment of QOL and concluded that EMST treatment is not a burden. Kuo and colleagues (12) found no differences in QOL after EMST using the Parkinson's Disease Questionairre-39 (PDQ-39), while Riboldazzi and colleagues (13) found significant improvement on the PDQ-39 after a 12-month protocol of respiratory strength training. Due to various methodologies, treatment interventions, and measurements, there is no consensus on the effects of respiratory strengthening protocols on QOL or adherence. Per available literature, there appears to be no studies that measure depression or anxiety with respect to EMST. Conversely, TS shows a positive impact on QOL (8, 9, 14), depression (15), and anxiety (16).

Despite these interventions showing improvement on respiratory physiological outcomes, adherence is an often omitted but crucial factor to consider in intervention trials. Adherence to treatment depends on how person-centered and individualized the participants feel the intervention is (17, 18). Regrettably, adherence to exercise interventions is especially low for people who are over 65 years and even lower for individuals diagnosed with a chronic condition like PD (19); up to 74% of PwPD reported lower adherence to their medication with depression being the strongest predictor of lower adherence (20). There remains sparce information on the individual factors that contribute to adherence to respiratory interventions.

Assessing self-reported outcomes may better inform individualized care by engaging the primary stakeholder (the PwPD) and lead to a more person-centered approach to care, improving adherence to treatment (18, 21). To gain the most thorough insight into these interventions, a mixed-methods research approach is warranted. The purpose of this study is to (1) quantitatively compare EMST and TS on measures of QOL, depression, and anxiety, (2) qualitatively understand the participant's perspectives on each intervention, and (3) integrate the datasets for a comprehensive understanding of EMST and TS to determine if they are acceptable and accessible options to increase treatment adherence. Because the literature shows more robust improvement in psychosocial measures after TS than EMST, it is hypothesized that post-intervention participant questionnaires will show more improvement in QOL, depression, and anxiety after TS. Qualitative analyses are expected to reveal themes that will provide a deeper understanding of participant's treatment experiences and preferences.

## Methods

### Design

**Figure 1:**
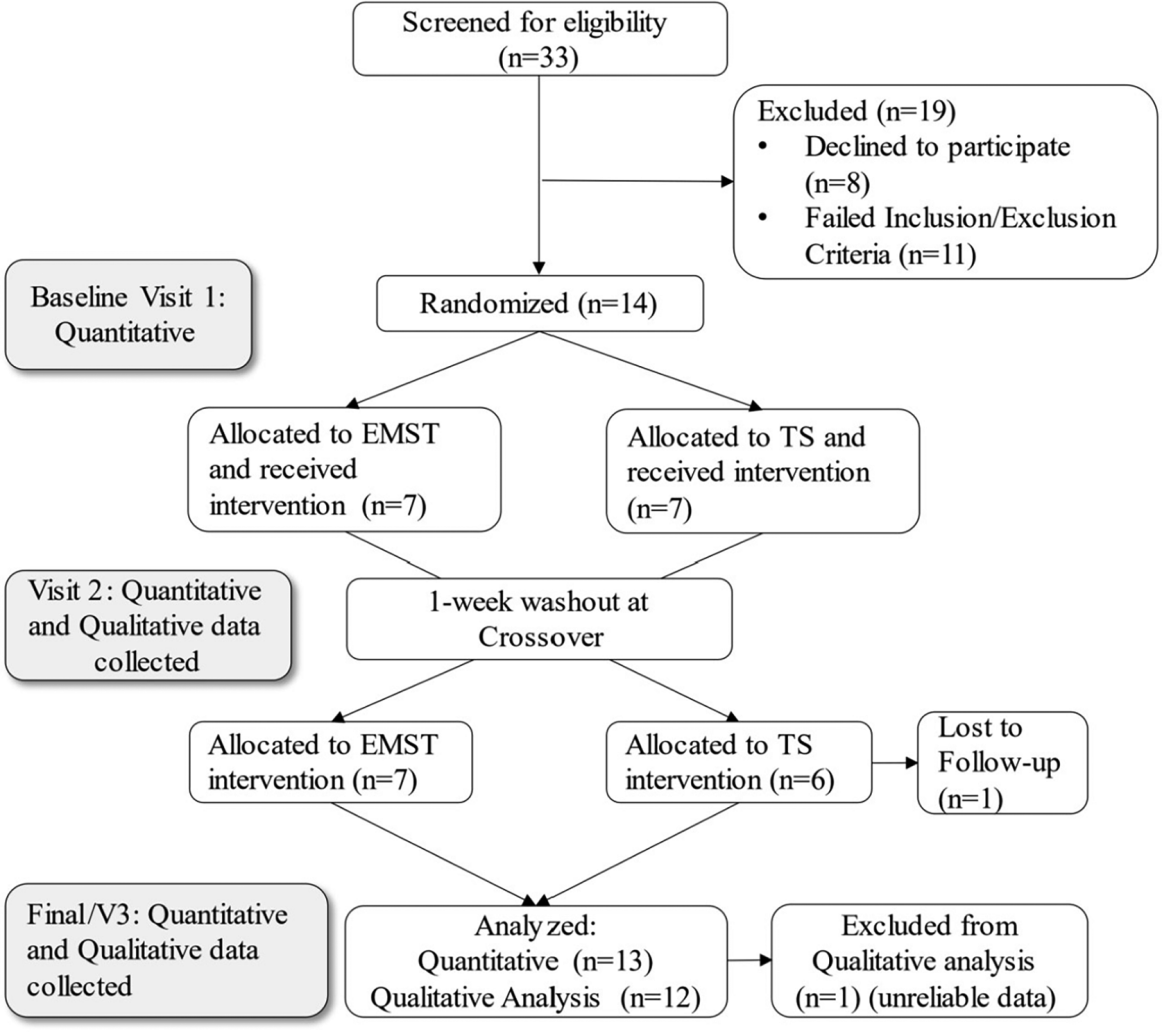
CONSORT diagram showing the flow of participants through the stages of the mixed methods intervantion design.

This study follows a pragmatist philosophical stance, which stems from the belief that the research questions (how EMST and TS compare on measures of QOL, depression, and anxiety, and what participant's perspectives are on each intervention's acceptability and accessibility) should guide the methodological approach (22). Thus, a mixed methods convergent design was used to utilize the strengths of both quantitative and qualitative analyses (23, 24). Qualitative data were embedded within a randomized crossover trial so participants could directly compare each intervention (Figure 1) (23, 25). The purpose of this design was for the qualitative results to provide a multi-layered understanding of the research question, as quantitative data alone may not provide a complete understanding of the complex nature of effectiveness and perceptions of the two interventions (23). Moreover, the purpose of using an embedded mixed methods intervention design is to understand the “transferability of the evidence” to determine if these interventions will be effective in the real world (25 p.407). More detail about each intervention is provided below.

### Participants

There are no established recommended sample sizes in mixed methods research, but a sample size of approximately 10 for phenomenological methodology has been suggested (24). Additionally, for research that aims to understand perceptions and experiences of a homogenous group (e.g., the PwPD in this study), twelve is recommended (26). Therefore, the sample size for this study was twelve.

Participants met the following inclusionary criteria: confirmed diagnosis of PD, between the ages of 55–85 years old, Hoehn and Yahr stage of I-III (mild-moderate), on stable medication for thirty days, ability to close and maintain lip seal around a mouthpiece, no cognitive impairment (Mini Mental State Exam ≤24), no symptoms of depression (Beck's Depression Inventory >18), no smoking in the past 5 years, no pulmonary diagnoses, no speech or music therapy within the past 6 months. During the baseline visit demographic information was collected (Table 1). No participants had a diagnosis of dysphagia. All participants provided written informed consent to participate in the study as approved by the Iowa State University Institutional Review Board.

**Table 1:** Group demographics.

	TS first	EMST first
Age (years)	69 ± 7	70 ± 7
Gender (% male)	42%	57%
Education (years)	17 ± 2	18 ± 4
Disease duration (years)	9 ± 7	8 ± 8
H&Y	2 ± .5	1.8 ± 0.40
MMSE	29 ± 1	29 ± 1
BDI*	12 ± 3	5 ± 3
MDS-UPDRS	58 ± 22	49 ± 19

Means and standard deviations for demographic measures and questionnaires. H&Y, Hoehn and Yahr scale; MMSE, mini-mental status exam; BDI, Beck's depression index; UPDRS, unified Parkinson's disease rating scale.

* *p* < .001.

### Interventions

#### Expiratory muscle strength training

Participants were provided with an EMST150 device and taught how to use it with an instructional video on the device's website (www.emst150.com). Resistance was set at 75% of their baseline maximum expiratory pressure. Participants were instructed to increase resistance by a quarter turn each week and use it for 5 days per week, 5 sets of 5 breaths, for four weeks as shown in previous studies (11, 27). Duration for each day's treatment was approximately 15–20 min.

#### Therapeutic singing

Participants were given a home-based therapeutic singing protocol to complete five times per week for four weeks via a private YouTube channel. Participants used one video for the duration of one week before switching to the next video, which progressed in difficulty. The TS protocol was led by a board-certified music therapist and based on protocols used in prior published research that took place once per week for 8 weeks (9, 14). Each weekly video was edited to match the frequency and duration of exercise as closely to the EMST protocol as possible. The TS intervention, which is approximately 25 min in duration and progressively required more effort each week, consists of a warm-up song with upper body movements to relax muscle stiffness and reduce rigidity, followed by vocal exercises, abdominal breathing exercises to increase breath control, and lastly singing three familiar songs with the goal of improving phrase length each week. These songs target breath control, vocal range, and articulation.

### Measures and data collection

Participants were recruited using purposive sampling. Participants completed both interventions in random order (TS or EMST first) which was determined through a random number generator. Participants completed three data collection visits: Baseline (V1), Visit 2 after the first intervention, and Visit 3 after the second intervention (see Figure 1). Two interviews were conducted with each participant (at V2 and V3) to ensure a quality sample of participants and support data saturation (details provided in the Qualitative Methods section below). One participant's qualitative data was excluded from analysis because of off-topic and unclear responses. Therefore, qualitative data from 12 participants were analyzed, and 13 participants' surveys were included in quantitative analysis (Figure 1). All data collections were completed in a private space either in the lab, at participants' homes, or a local community center if they lived too far away to travel to the lab.

#### Quantitative methods

Four questionnaires were used. The PDQ-39 (28) is a valid and reliable measure of QOL that uses a 39-item questionnaire to assess difficulties that PwPD experience across 8 dimensions of daily living. The Geriatric Depression Scale (GDS) (29) is a valid and reliable depression scale for PwPD. Five or more points on this scale suggests depression, and a lower score indicates lower depression. The Parkinson's Anxiety Scale (PAS) (30) is an acceptable, reliable and valid for assessing anxiety in PwPD. A lower score indicates less anxiety. A survey after treatment (SaT) was developed as a quantitative measure of acceptibility and consisted of six questions on a 5-point Likert-scale to obtain information regarding treatment fidelity and quantify perceptions of each treatment post-intervention, with a low score being a more positive experience. Finally, participants kept daily treatment logs which enabled treatment fidelity to be assessed and quantified. Participation data from these logs were inspected to ensure adequate engagement in the treatment.

#### Qualitative methods

Semi-structured interviews were conducted by the first author at V2 and V3 to obtain the participants' perspective of their experience for both interventions and took approximately 20–30 min. Interview questions were open-ended and focused on preferences and perceptions of both interventions (Table 2). Questions focused on understanding differential experiences during EMST and TS, namely benefits and/or burdens, and how likely they were to continue either treatment after the conclusion of the study.

**Table 2:** Questions used to guide the semi-structured interviews.

Describe your overall impressions about the intervention you just finished.
Describe any changes in your health regarding:
Breathing:
Coughing:
Swallowing:
During the last four weeks, what kinds of changes in your emotional or mental well-being did you experience?
Describe any burdens experienced during this intervention.
ON FINAL VISIT ONLY: Please tell me your perceptions and opinions of both interventions; compare/contrast your experience in both.
What else would you like to share with us about your experience in the intervention?

### Data analyses

#### Quantitative analysis

To examine if there were any baseline differences between the two groups, independent sample *t*-tests were conducted to compare age, education, disease duration, MMSE, BDI and UPDRS scores (Table 1). Scores for all surveys were totaled for each timepoint for a main score on the PDQ-39, PAS, and GDS, and independent *t*-test were completed for the baseline (V1) scores to test baseline differences between groups. To test the main hypotheses, a 2 (TS first or EMST first) by 3 (Baseline/V1, V2, and V3) repeated measures ANOVA for each questionnaire was completed. *Post hoc* comparisons were completed using Bonferroni correction when appropriate. Scores on the SaT after each intervention were pooled and analyzed using Wilcoxon rank sum tests to test the within-condition differences for each of the six constructs. Effect sizes were calculated using partial eta squared. Fidelity logs were tallied for completion rate percentage.

#### Qualitative analysis

The interviews were transcribed verbatim by a professional service (www.rev.com). Complete transcripts were entered into MAXQDA (VERBI Software) and analyzed using theoretical thematic analysis (31). The transcripts were checked for accuracy, and segments were assigned a descriptive code using inductive coding (24). Data were grouped into themes that were identified from recurring responses or ideas within the transcripts (31). The embedded qualitative data from the SaT were grouped into codes using deductive structural coding (24).

The first author conducted all data collection and analyses. To show transparency in these findings, it is essential to understand the ways in which the qualitative analyses were influenced by the researcher's own background and demographics. The first author (J.B.) is a white woman, highly educated, with professional and personal experience with PwPD. This background shaped the lens through which she conducted the interviews and interpreted the findings. To increase trustworthiness and credibility, an audit trail was established through notes taken during data collection as well as via memos in the analysis software. Member-checking of synthesized data was offered to three participants who reviewed the findings to ensure that their own experiences were accurately and reliably captured within the final results.

#### Integration of the data

Both datasets were compared and triangulated to determine to what extent the findings converge, diverge, or expand to lead to main insights into the accessibility and acceptability of the interventions (24). A joint display of the SaT quantitative and qualitative responses was created to inspect differences between the participants' perceptions and experiences of both interventions (Table 3).

**Table 3:** Joint display of participant experiences.

SaT question	Test statistic (*t, p*)	Participant experiences	Example quotes
How easy to use intervention	−1.753, .080*	Knowing the lyrics to songs would make TS easier Portability of EMST makes it easier Scheduling time to do TS was more difficult Difficult to know which way to adjust the EMST device.	TS: “The only thing I would do is at least let me see the words once written down so I can remember them.” (11) EMST: “The tube was easier because you didn't have to worry about making noise for other people to hear.” (14)
How motivated to complete intervention	−1.058, .290	The nature of being in a research study was motivating Experiencing symptom improvement was motivating Flexibility of using EMST a motivator Seeing others in TS videos was motivating.	TS: “I think it was a cool way to address muscle exercises…If you were to tell me that you have to go outside and go boom, boom for five minutes, I probably wouldn't do it. But doing it in the context of what it was doing for your throat and all your facial muscles really made me..Smiling. So that was probably a way to motivate.” (11) EMST: Very. The ability to take it with me when I traveled was key, and flexibility to choose my five days within the week was helpful. (14)
How likely to continue intervention	−.288, .773	Likelihood of continuing depends on whether they saw benefits. Would continue or return to the intervention if symptoms become worse Use interventions for maintaining current functioning.	TS: “As the future goes on and everything, I may think back, well, maybe I … should be singing better, trying to do a better job of singing in places where I could sing.” (12) EMST: “I might … just to do it as an exercise just like you do other exercises to … continue to keep it where it's at, maybe improve a little…Yeah, maintain. I think a maintaining level rather than improving level.” (07)
Level of social engagement	−1.265, .206	Social engagement was not a meaningful benefit for either intervention Felt a part of the TS group even though it was done individually Improved symptoms (voice, breath, cough) led to more social engagement.	TS: “I've been getting like rigidity in my face, and singing would loosen my face muscles up, so it was easier for me to talk to people so in that perspective my social engagement improved. I would agree with that. I didn't assess the same thing when I was blowing through the tube.” (14) EMST: “I would say I would agree because I'm not coughing as much.” (07)
How enjoyable was intervention	−.690, .490	No reports of enjoying the EMST intervention even if it was beneficial/easy Enjoyability depended on prior singing experience and song choice preferences Group TS would be more enjoyable Singing in front of others was an issue for non-singers.	TS: “Overall, I know there's benefit to it, but as far as actually enjoying and saying that I want to do it, it's not there.” (01) EMST: “It wasn't something I look forward to. Oh, goody, this’ll be a riot while having a blast, but it wasn't unpleasant where I dreaded it or something.” (05)
How helpful was intervention	−.965, .335	TS was helpful in a more comprehensive way, such as posture, memory, facial musculature EMST was helpful in expected ways (vocal quality and volume, breathing strength).	TS: “I'm going to say somewhat helpful because it reinforced some things that… are just good practice; the posture stuff and good breathing.” (05) EMST: “I think it helped me. It just helped me. In my job, I do a lot of talking, and it seemed like I had a little more… I didn't get as tired as much, and I had a little more voice quality to talk through a little louder.” (08)

Survey after Treatment (SaT) question topic. Test statistic (*t*) for differences in total scores of SaT after each intervention regardless of treatment order, significance (*p*) value for Wilcoxon Ranked Tests. No significant differences were found between interventions for any question; “Easy” did not reach significance (*p* = .080), but showed an interesting finding in favor of EMST indicated with (*). Lower scores indicate better outcomes. Example quotes to expand knowledge from the SaT. EMST, expiratory muscle strength training, TS, therapeutic singing.

## Results

### Quantitative results

There were no significant differences between groups at baseline for age, MMSE, education level, UPDRS scores, or disease duration. A significant difference was found between groups for BDI score (Table 1), with TS-first group having a higher mean (11.58) compared to the EMST-fist group (4.50), but neither group met the criteria for clinical depression. There were no significant differences in PDQ-39 (*p* = .155), PAS (*p* = .257), or GDS (*p* = .700) at baseline.

**Figure 2:**
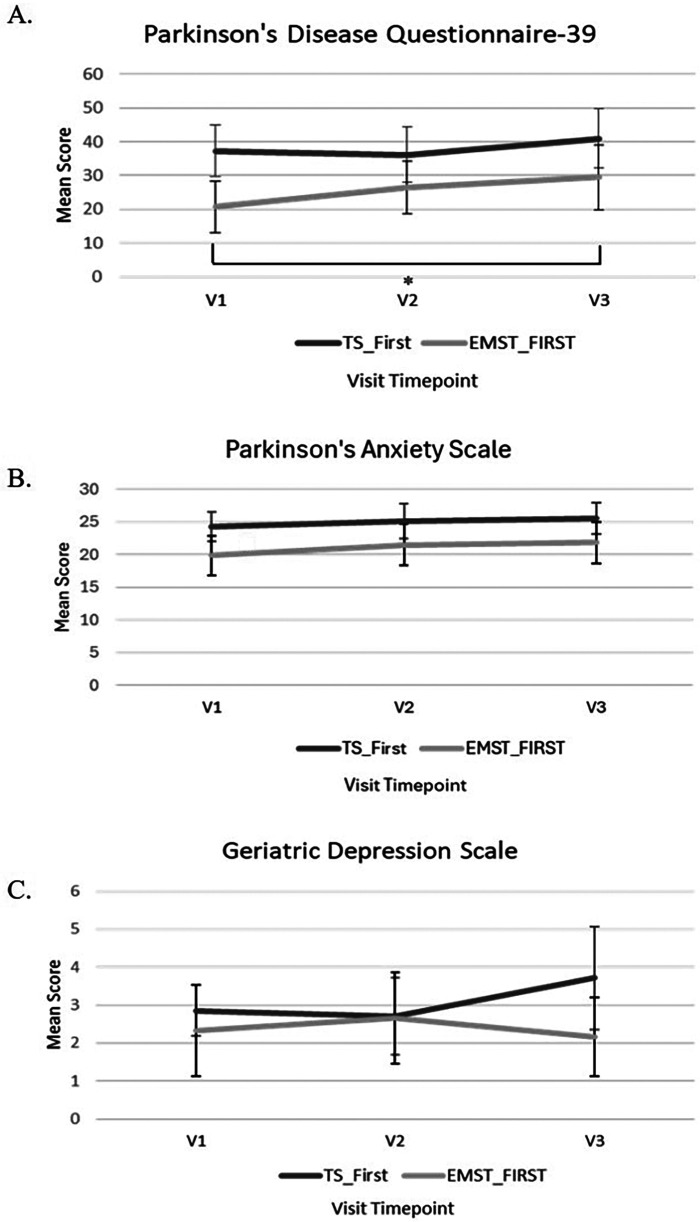
**(A)** Mean scores and standards errors for the Parkinson’s disease questionnaire-39 (PDQ-39) after each visit timepoint. Significant differences found between V1 and V3 (*p = 0.010) with pairwise comparisons and Bonferroni correction for multiple test. **(B)** Mean scores for the Parkinson’s anxiety scale after each visit timepoint. **(C)** Mean scores for the geriatric depression scale after each visit timepoint. Lower scores reflect better outcomes.

Figure 2A shows the means and standard errors for the PDQ-39 for each intervention at each timepoint. In general, the TS-first group had consistently higher scores on the PDQ-39 than the EMST-first group. Lower scores indicate higher QOL. The scores for TS-first group did not change over the course of the study, but the EMST-first group showed an increase in scores over both treatments. Statistical analysis revealed a significant main effect of time [*F*(2,10) = 4.744, *p* = .04, partial *η*^2^ = 0.49], but not for group [*F*(1,5) = 3.837, *p* = .10, partial *η*^2^ = 0.43], or time × group interaction [*F*(2,10) = 1.357, *p* = .30, partial *η*^2^ = 0.21]. *Post hoc* analyses (Bonferroni corrected, *p* < 0.013) using paired *t*-tests for the main effect of time revealed a significant increase (*p* = 0.010) in PDQ-39 scores for comparisons between V1-V3 only. No other comparisons reached significance (*p* > 0.05).

Results from the PAS are shown in Figure 2B. In general, the TS-first group rated their anxiety higher than EMST across all three timepoints. However, statistical analysis revealed no significant main effect of time [*F*(2,10) = .724, *p* = .51, partial *η*^2^ = 0.17], group [*F*(1,5) = 5.695, *p* = .06, partial *η*^2^ = 0.53], or time × group interaction [*F*(2,10) = .12, *p* = .88, partial *η*^2^ = 0.23].

Results from the GDS are shown in Figure 2C. In general, both the TS-first and EMST-first groups had roughly equivalent scores at V1 and V2, but after crossover, scores increased slightly (indicating more depression) after EMST and decreased slightly after TS (indicating less depression). However, statistical analysis revealed no significant main effect of time [*F*(2,10) = .354, *p* = .71, partial *η*^2^ = 0.06], group [*F*(1,5) = .764, *p* = .42, partial *η*^2^ = 0.13], or time × group interaction [*F*(2,10) = 2.818, *p* = .11, partial *η*^2^ = 0.36].

Results for the SaT are shown in Table 3. In general, the pooled scores after TS were higher than after EMST (lower scores indicate more positive responses). However, Wilcoxon rank sum tests for each of the six questions on the SaT revealed no significant differences between either intervention; although not statistically significant, ease of use showed interesting results favoring EMST (*p* = .080). Completion rates on self-reported fidelity logs were 94% for TS and 99% for EMST.

### Qualitative results

The qualitative analyses resulted in five main themes (Benefits, Accessibility, Acceptability, Advice/Feedback, and Preference), and organized into three ideas (Benefits from interventions, Perceptions of interventions, and Comparison of interventions). The number in parentheses is the participant's ID number.

#### Benefits from interventions

There were mixed results with respect to how participants' symptoms improved because of the interventions (*Theme 1*). Participants reported improved breath strength and vocal volume after both interventions. Four participants reported improvement with swallowing after EMST, mostly with taking pills or eating certain food textures. Other symptoms that improved with TS were memory skills from remembering all the singing techniques and lyrics, as well as social benefits from feeling as if they were in a group setting (even though they completed it individually). One participant expressed that both interventions were helpful: “I think they both helped my breathing. I think back to day one when I started with EMST and how at the end of the fourth week I had gone so much further, and it's the same way with the singing. When I first started singing, I couldn't go very far, and my enunciation wasn't very good. I think they both severely helped me a lot” (02).

#### Perceptions of interventions

Participants' overall opinions and feedback about the intervention ranged from very positive to negative regarding accessibility (*Theme 2*) and acceptability (*Theme 3*). Participants had more polarized feedback on TS: either they enjoyed it or did not. An influencing factor for this was the participant's comfort level with singing, whether they saw any beneficial results, and how easy or difficult they found the intervention to be. Overall, participants found EMST to be more accessible to use than TS because it does not produce noise and is more portable (they could use it anywhere). With TS, participants had to consider their environment to not disturb their partners, or to limit embarrassment if they were uncomfortable singing. For example, one participant expressed that TS was “somewhat difficult because … the logistics of it being online, and I was all by myself. I'd be singing, and I knew we were staying in a condo for four weeks, and all the people around me, I'm sure could hear me singing” (13). Another influential factor that led to negative reviews of the TS were the song selection and not knowing the lyrics/songs in advance. In general, while participants did not report enjoying EMST *per se*, they were not burdened by it.

Participants gave advice and feedback regarding how to make the interventions better (*Theme 4*). The recurring theme for TS was diversifying the song selection to make the song choices more contemporary and/or recognizable and providing lyrics at the beginning of the intervention so they could more accurately engage. There was no feedback regarding the intensity or frequency of either intervention. For EMST, a recommendation was for there to be a clearer way to know which way to turn the device to increase the pressure threshold, as it caused confusion for some.

#### Comparisons of interventions

The crossover study design allowed for direct comparisons between both interventions regarding preference (*Theme 5*), as well as if they will continue to use intervention. Ten out of twelve participants evenly expressed a clear intervention preference for TS (*n* = 5) and EMST (*n* = 5). Reasons they did or did not complete the entire protocol (adherence) aligned with how accessible and acceptable the interventions were deemed to be. For example, one participant expressed the following sentiment about continuing TS: “Well, my voice is not very good, but I think it'll help me in the long run. Because as my disease gets worse and worse, I'll probably go back more and more to the singing part to keep my voice going” (09).

### Integration of quantitative and qualitative results

The qualitative findings reflect what was found in the quantitative data, that preferences and perceptions of the interventions were roughly equivalent. Together, these data provide a comprehensive picture to expand our knowledge of the interventions (Table 3). There were no significant differences in the PAS or GDS scales, and this is also reflected in the qualitative data: there were no themes that emerged regarding anxiety or depression, or how these factors might have influenced their experiences. However, there was a significant difference in participants' self-report score of QOL on the PDQ-39, which worsened over the course of the study (from V1 to V3) indicating that regardless of perceptions of the interventions, participants rated their QOL as lower as time progressed. This is a divergence from the qualitative analyses; no qualitative themes emerged regarding a decrease in overall QOL resulting from the interventions. Interpretations of this divergence are discussed below.

## Discussion

This study aimed to compare two respiratory control interventions (EMST and TS) on measures of QOL, depression, and anxiety in PwPD, and to understand participants' perspectives on each intervention and how their experiences impact treatment adherence and continuation. Because previous findings show more improvement in psychosocial measures after TS than EMST, it was hypothesized that there would be a greater improvement in QOL, depression, and anxiety after TS. This was not confirmed as results revealed no differences between interventions. Qualitative analyses provided important context to the quantitative findings, and allowed a deeper understanding of participants' treatment experiences and preferences.

Findings indicate that the EMST and TS interventions did not differ significantly from each other with respect to QOL, depression, and anxiety, which is in contrast to the study hypothesis. One possible reason the hypothesis was not supported is that mood states are highly complex and influenced by psychosocial factors like social support, coping style, and personality traits, which contribute to varied perceptions (32).

Scores on the PDQ-39 increased (worsened) significantly from baseline to V3 for the EMST-first group, but not the TS-first group. This finding is congruent with the literature in that PDQ-39 scores worsen longitudinally because demographic factors and baseline disease states that affect QOL and are difficult to modify (33). Health-related QOL factors for PwPD are highly heterogenous depending on the PD phenotype (i.e., early vs. older, disease progression rate, and motor-dominancy) (34). The participants in this study were relatively high functioning with no reports of respiratory or cough concerns. It is possible that this sample was not aware of potential impending non-motor symptoms that were mentioned in the questionnaire. Thus, participation in this study may have emphasized negative symptomology, resulting in reduced feelings of hope and well-being, which are important factors in QOL in PwPD (35). However, the qualitative findings in this study did not reflect any themes about QOL, either positive or negative.

Results from the GDS and PAS showed no significant changes over time or between treatment interventions. Again, previous literature shows decreased feelings of depression and anxiety after singing (15, 16). To our knowledge, this is the first study to assess scales of anxiety and depression for PwPD after EMST. In a sample of individuals more progressed in PD symptomology, as well as a larger sample size, differences may be detected in self-perceptions of anxiety and depression. Another possible reason that no differences were found between interventions is because the TS-first group had a significantly higher baseline level of depression than the EMST-first group, making comparisons of the treatments difficult to establish. Yet, the qualitative data support the lack quantitative significance found; no themes emerged regarding a change in anxiety or depression associated with either intervention.

Participants' perspectives regarding each intervention and how these perceptions affect treatment adherence and continuation was primarily assessed with the SaT and simultaneous qualitative interview. Quantitative analysis of the summed scores after each intervention did not reveal significant differences on any construct, and qualitative analyses indicate an equal split in preferences for interventions. Furthermore, ease of use was the only construct that showed interesting statistical results that were also echoed in the qualitative analyses, with participants generally expressing that EMST was easier to use than TS, even if they expressed a preference for TS. As for which intervention they were more likely to continue, results were divided along individual preferences as well. Qualitative data suggest that for those who would continue TS, it would most likely not be in the pre-recorded format used in this study, but rather incorporated into their daily routine. Those who said they would continue EMST said they would do so when their respiratory, swallow, or cough symptoms worsened. Finally, adherence to both treatments was very high (≥94%). This high compliance may indicate that both treatment modalities were acceptable and accessible, and treatment preferences align with individual differences.

This study aimed to directly compare two evidence-based respiratory interventions, not determine their effectiveness as has been done in prior studies. Thus, these findings can be used to inform clinical practice for PwPD. For TS, singing in a group setting, rather than individually, appears to facilitate more comprehensive benefits. More familiar song choices and provision of lyrics once at the beginning of the intervention are two ways that may enhance the TS protocol. For EMST, a clearer way to indicate adjusting the threshold level would make it easier to use, and weekly contact (either in-person or via video call) to ensure proper recalibration is important for accurate use. The integrated data analyses can be used to infer clinical implications, such as the importance of considering individual differences and preferences when recommending a treatment protocol, leading to more effective person-centered care. Finally, clinicians may consider using both TS and EMST interventions to address a wider range of PD respiratory symptoms than one treatment alone can render.

This study is not without limitations. Measuring complex constructs like QOL, depression, and anxiety are highly contextual. While every effort was made to complete assessments at the same time after taking medication, it was not always possible. Life events, health status, travel, and other pertinent variables between assessment visits might have influenced perceptions and experiences of the interventions, and while a questionnaire was used to explore these factors, quantitative analyses did not control for their influence on outcome measures but rather used to uncover potential variability in the results. Also, the limited diversity in demographics makes it difficult to generalize these results to a broader population, including those with more progressed PD.

Future studies may detect more significant functional changes in QOL, depression, and anxiety in those with more progressed PD symptomology, including a dysphagia or pulmonary diagnoses, and utilize self-perception surveys that are specific to that diagnosis.

## Conclusions

There is limited research on participants’ experiences with respiratory interventions for PwPD. This research helps to fill that gap by elucidating the acceptability, accessibility, and overall perceptions of TS and EMST. Results describe ways to involve PwPD in these interventions more effectively, thus improving care and potentially fostering sustained engagement.

## Data Availability

The raw data supporting the conclusions of this article will be made available by the authors, without undue reservation.
